# The Shortage of Platinum

**Published:** 1916-02

**Authors:** 


### THE SHORTAGE OF PLATINUM.

Unfortunately for those with a taste for speculation in "war brides" there is no opportunity for trading in the shares of any platinum companies.

The price of the metal has advanced, since the beginning of the war, from $40 to close to $100 an ounce, with prospects for its going higher. Manufacturing jewelers are commonly supposed to be the chief sufferers from the jump in price. A fair sized jewelry firm making a general commercial line of jewelry will use from 200 to 250 ounces of platinum in a year. Contrasted with this is the recent purchase by the Du Pont people of 112 pounds of platinum at prices ranging from $55 to $80 an ounce, and representing an outlay of upwards of $85,000. At $100 an ounce, 112 pounds would be worth $134,400. Agents of the company have instructions to purchase any platinum in sight. The platinum is used in the laboratories for making crucibles and other receptacles for acids, which would eat through any other metal. When the war is over, the price of platinum may be expected to fall to normal, which is about $40 an ounce.

One of the most important uses of platinum is as a catalyzer in what is technically known as "contact mass" in the manufacture of fuming sulphuric acid and sulphur trioxide. There are several kinds of "contact mass," the two most used in this country being on asbestos or magnesium sulphate bases. The mass is made by soaking the base in solutions containing platinum chloride and afterwards heating it. This treatment results in a more or less complete distribution of very fine particles of metallic platinum throughout the mass. Some "contact mass" contains as much as 7 to 8 per cent. by weight of platinum, and other manufacturers make a mass containing as little as 0.2 per cent. metallic platinum by weight.

It is impossible at this time to state the quantity of platinum used in the sulphuric-acid industry in this country, but it is believed that the loss of platinum in well-regulated practice is very small. The platinum does not enter into the chemical reactions, but rather acts as an exciter in the formation of sulphur trioxide, which, on combination with water, yields sulphuric acid.

Platinum dishes and utensils of many kinds are still a necessity in chemical laboratories and many chemical industries, but the use of this metal in the electrical industry is each year becoming less. In the manufacture of incandescent lamps a large quantity of platinum was formerly used, but at present wires made of nickel-chromium alloys or metallic tungsten or metallic molybdenum are used. The resistance wires of electric furnaces and heaters at one time contained considerable platinum, but metallic molybdenum has now to a considerable extent replaced the more expensive metal. Formerly the electrical ignition points of gas engines were made of platinum, but it is believed that most of the spark-plug points now used are made of metallic tungsten.

The dental industry once used a large quantity of platinum in making artificial teeth. It is understood that recently metallic molybdenum plated with platinum is widely used rather than pure platinum.

The art of platinum plating has now been perfected to so remarkable a degree that jewelers are no longer under the necessity of using so much platinum as formerly in making jewelry, though platinum is still considered the best setting for precious gems. This use of platinum, however, is somewhat subject to the reigning fashion. Of interest in this connection is a bill which was prepared by the National Jewelers' Board of Trade of New York early in 1914 for presentation to the New York Legislature, making it a misdemeanor to mark inferior jewelry "platinum," "pure platinum," or "solid platinum" unless it is

0.950 fine or contains 95 per cent. platinum group metals.

A somewhat similar law was passed by the Swiss Federal Council, to be effective March 1, 1914. This law provides that "upon request of the manufacturer, seller, or purchaser, articles having a minimum platinum content of 95 per cent will receive the official stamp of an Alpine goat."

In Germany, it is understood, an alloy of rhodium and palladium, metals of the platinum group, is being used in the manufacture of jewelry.

Many manufacturers in Maiden Lane remember when they bought the metal for $5 to $10 an ounce, but that was just after the civil war. About twenty-five years ago Maiden Lane had a big demand for watch chains made of alternate links of gold and platinum, but this fad died out and platinum was thereafter not used by jewelers until they found it better than gold in mounting diamonds.

In 1905 pure platinum was down to $18.50 an ounce. Early in 1906 the consumption began to increase and the price advanced until it was doubled in December of that year, and continued until August 1908, when the level of 1905 was again reached. In January, 1909, the price had again advanced to $24.25 an ounce and during the year increased to $29,50. During 1910 the price advanced to $38.75 an ounce and by December, 1911, the metal was selling at $46. The quotation remained around that price until March of this year, when it dropped to $42. In August last platinum was worth $41 and during September jumped to $51. October quotations advanced to $60 an ounce and today the man who gets it near $100 is getting a bargain.

Crude platinum is on the market in the shape of small nuggets, ranging in weight from 100 mg. down. It may be divided roughly into two portions, the insoluble and the soluble in aqua regia. The insoluble includes osmiridium as its only valuble constituent, while the soluble includes platinum, iridium, palladium, rhodium, and gold. It is desired to determine each of the above metals with accuracy.

Platinum as found in the placers of the western states usually occurs as small scales or flakes, but in some placers it occurs as irregular nuggets. The distinctly metallic mineral has a color ranging from silvery white to steel-gray, its shade depending on the quantity of impurities present. In some placer deposits the grains of platinum are coated with a dark film and somewhat resemble grains of ilmenite or magnetite, from which, however, they are separated by careful washing, as platinum has a specific gravity equal to or greater than gold, and so stays in the pan with the gold.

Up till the war the French company (Compagnie Anonyme) always had a predominating interest in the platinum business, which practically amounted to a monopoly, having entered into agreement with the chief big producers for the exclusive purchase of their ware, besides being large producers themselves. Since the outbreak of the war, however, the French company have naturally been unable to meet their obligations of purchase, with the result that their former clients have been obliged to resort to the only way out of the difficulty, viz., mortgage their ore with the banks in Russia. Considerable stocks are lying mortgaged at the various banks (not less than 45,000 ounces). The output for the current year is estimated to be very much less than for former years.

The recently published rumor that platinum has been discovered in Spain was confirmed in November by Professor Orueta. The latter is a Spanish mining engineer and was designated by the Instituto Geologico (Geological Society) to examine the Ronda Mountain Range for possible mineral deposits. He has made an address before the Instituto de Ingenieros de Espana (Society of Civil Engineers of Spain) in Madrid, in which he sets forth his labors, and their results. He states that he has discovered platinum deposits of greater extent and richness than those of the Ural Mountains in Russia, which furnish about 90 per cent. of the world's supply.

By royal decree, published November 17th in the Gaceta de Madrid, official organ of the Spanish government, the territorial limits of the mines are defined. Concessions to exploit the mines are obtainable from the Spanish government, and these concessions are granted to foreigners as well as to Spanish subjects. Applications for such concession should be made to Senor Ministro de Fomento, Madrid, Spain. The deposits of platinum are said to be the richest in the world.

Now that they are not busy as they were, jewelry manufacturers who use platinum in their wares are coming to the conclusion that the recent sharp advances in the price of this metal were not altogether warranted by the law of supply and demand. Since the manufacturing demand has fallen off prices have begun to recede, quotations ranging from $90 to $96 an ounce, with the latter figure the more generally asked. About two weeks ago the metal was quoted all the way up to $106 an ounce. In addition to the lowered price, platinum is being offered from so many sources that it is said to be difficult to believe it is as scarce as recently reported, in spite of the embargo, which leaves manipulation as the reason for the recent advance. Manufacturers are reported to have decided to make up as little stock goods as possible with platinum at its present value, and some have turned to white gold for use in their less expensive pieces. For the finer pieces, however, platinum will be used regardless of its cost, as no other known metal is so suitable for the jewelers' purposes —*The American Jeweler*, January, 1916.

